# Inhibition of STAT3 dimerization and acetylation by garcinol suppresses the growth of human hepatocellular carcinoma *in vitro* and *in vivo*

**DOI:** 10.1186/1476-4598-13-66

**Published:** 2014-03-21

**Authors:** Gautam Sethi, Snehajyoti Chatterjee, Peramaiyan Rajendran, Feng Li, Muthu K Shanmugam, Kwong Fai Wong, Alan Prem Kumar, Parijat Senapati, Amit K Behera, Kam Man Hui, Jeelan Basha, Nagashayana Natesh, John M Luk, Tapas K Kundu

**Affiliations:** 1 Department of Pharmacology, Yong Loo Lin School of Medicine, National University of Singapore, Singapore 117597, Singapore; 2 Cancer Science Institute of Singapore, National University of Singapore, Singapore 117456, Singapore; 3 Jawaharlal Nehru Centre for Advanced Scientific Research, Molecular Biology and Genetics Unit, Transcription and Disease Laboratory, Jakkur P.O., Bangalore 560064, India; 4 School of Biomedical Sciences, Faculty of Health Sciences, Curtin University, Bentley, WA 6009, Australia; 5 Department of Biological Sciences, University of North Texas, Denton, TX 76203, USA; 6 Division of Cellular and Molecular Research, Humphrey Oei Institute of Cancer Research, National Cancer Centre, Singapore 169610, Singapore; 7 Institute of Molecular and Cell Biology, 61 Biopolis Drive, Proteos, Singapore 138673, Singapore; 8 Central Government Health Scheme Dispensary, No. 3, Basavanagudi, Bangalore, India

**Keywords:** HCC, STAT3, Acetylation, Garcinol, Apoptosis

## Abstract

**Background:**

Constitutive activation of signal transducer and activator of transcription 3 (STAT3) has been linked with proliferation, survival, invasion and angiogenesis of a variety of human cancer cells, including hepatocellular carcinoma (HCC). Thus, novel agents that can suppress STAT3 activation have potential for both prevention and treatment of HCC. Here we report, garcinol, a polyisoprenylated benzophenone, could suppress STAT3 activation in HCC cell lines and in xenografted tumor of HCC in nude mice model.

**Experimental design:**

Different HCC cell lines have been treated with garcinol and the inhibition of STAT3 activation, dimerization and acetylation have been checked by immunoblotting, immuno-fluorescence, and DNA binding assays. Xenografted tumor model has been generated in nude mice using HCC cell line and effect of garcinol in the inhibition of tumor growth has been investigated.

**Results:**

Garcinol could inhibit both constitutive and interleukin (IL-6) inducible STAT3 activation in HCC cells. Computational modeling showed that garcinol could bind to the SH2 domain of STAT3 and suppress its dimerization *in vitro*. Being an acetyltransferase inhibitor, garcinol also inhibits STAT3 acetylation and thus impairs its DNA binding ability. The inhibition of STAT3 activation by garcinol led to the suppression of expression of various genes involved in proliferation, survival, and angiogenesis. It also suppressed proliferation and induced substantial apoptosis in HCC cells. Remarkably, garcinol inhibited the growth of human HCC xenograft tumors in athymic nu/nu mice, through the inhibition of STAT3 activation.

**Conclusion:**

Overall, our results suggest that garcinol exerts its anti-proliferative and pro-apoptotic effects through suppression of STAT3 signaling in HCC both *in vitro* and *in vivo*.

## Background

Hepatocellular carcinoma (HCC) is the most frequent subtype of liver cancer and third leading cause of mortality worldwide, which results from poor prognosis [1-3]. More than 80% of HCC patients are presented at advanced stages of disease where surgical resection is difficult and other treatments such as chemotherapy are primarily palliative [4,5]. Thus, a great challenge lies ahead in identifying novel treatment regimens to overcome chemoresistance and prolong the life of HCC patients.

The family of STAT signaling proteins, consisting of seven members, is involved in important cell signaling events especially cytokine induced signal transduction pathway [6,7]. Among the various STAT family members, STAT3 has been most closely linked to tumorigenesis [8,9]. Persistent activation of STAT3 has been frequently observed in many kinds of tumors [8,9] including HCC [10-12] and this constitutively active STAT3 is thought to contribute to the process of oncogenesis by modulating the expression of various genes required for tumor cell survival (e.g., *Bcl-xL, Mcl-1, survivin*), proliferation (e.g., *cyclin D1, c-Myc*), and angiogenesis (e.g., vascular endothelial growth factor [*VEGF*]) as well as metastasis [8]. Thus, the targeted inhibition of STAT3 signaling pathway may provide significant therapeutic benefits to HCC patients.

Upon activation by cytokines (like IL-6) or growth factors (such as EGF, PDGF), STAT3 undergoes phosphorylation-induced homodimerization or heterodimerization, which in turn results in the nuclear translocation, DNA binding, and subsequent modulation of gene transcription. STAT3 phosphorylation can be mediated through the activation of upstream non–receptor protein tyrosine kinase family of Janus-like kinase (JAKs), and c-Src kinase [6]. STAT3 also gets acetylated by p300 at lysine 685 which is essential to form stable dimers [13]. Acetylated STAT3 binds strongly to the DNA, enhances transcription of genes essential for cell growth and thus contributes towards tumorigenesis. Acetylation of STAT3 is elevated in tumors and contributes towards tumor progression by inducing DNA methylation at the promoters of tumor suppressor genes [14]. Thus, targeting acetylation of STAT3 using small molecule approach could be a potential means for chemoprevention and cancer therapy.

In the present report, we analyzed the effect of garcinol, a natural polyisoprenylated benzophenone, isolated from the dried rind of the fruit *Garcinia indica*, which has attracted great attention of late for its potential anti-cancer effects [15-17]. Garcinol is a potent inhibitor of acetyltransferase activity of p300 and PCAF and inhibits transcription from chromatin template [15]. Garcinol has been reported to inhibit the proliferation and induce apoptosis in different tumor cells *in vitro*[16-19]*,* and also exhibit potent anti-oxidant, anti-inflammatory and bactericidal activities [20-22]. The ability of garcinol to modulate the expression of pro-inflammatory cytokines, inducible nitric oxide synthase (iNOS) and cyclooxygenase-2, NF-κB, STAT3, Akt, FAK, death receptors, nicotinic receptors, cyclin D3, and histone acetyltransferases (p300 and PCAF) has been reported [23-28]. However, the potential anti-cancer effects of garcinol in HCC cancer model and in the context of STAT3/JAK2 signaling cascade as well as STAT3 acetylation have not been investigated yet.

Because of the critical role of STAT3 in HCC survival, proliferation, invasion, and angiogenesis [29,30], we investigated whether garcinol can mediate its anti-proliferative and pro-apoptotic effects in HCC cells through the suppression of the STAT3 pathway. We found that garcinol can indeed suppress both constitutive as well as inducible STAT3 activation through blocking its dimerization, nuclear transport and DNA binding by inhibiting its phosphorylation and acetylation. This inhibition decreased cell survival and downregulated expression of proliferative, anti-apoptotic and angiogenic gene products, leading to suppression of proliferation and the induction of apoptosis in HCC cells. Garcinol also inhibited the growth of human HCC cells in a xenograft mouse model and modulated the activation of STAT3 in the tumor tissues.

## Results

**Figure 1:**
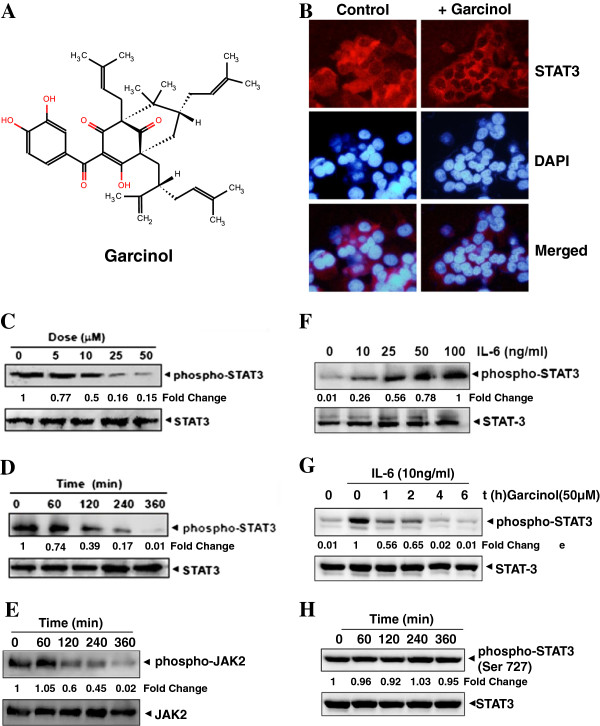
**Garcinol reduces the constitutively active form of *STAT3. *(A)** The chemical structure of garcinol. **(B)** Garcinol causes inhibition of translocation of STAT3 to the nucleus in C3A cells. **(C)** Garcinol suppresses phospho-STAT3 (Tyr ^705^) levels in a dose dependent manner. C3A cells (2×10^6^/ml) were treated with the indicated concentrations of garcinol for 6 h, and the whole cell extract was subjected to western blotting analysis using phospho-STAT3 antibody. **(D)** Garcinol suppresses phospho-STAT3 levels in a time-dependent manner. C3A cells (2×10^6^/ml) were treated with the 50 μM garcinol for the indicated times, after which western blotting was performed as described for panel C. The same blots were stripped and reprobed with STAT3 antibody to verify equal protein loading. **(E)** C3A cells (2×10^6^/ml) were treated with the 50 μM garcinol for the indicated times, after which western blotting was performed against phospho-JAK2 antibody. The same blots were stripped and reprobed with JAK2 antibody to verify equal protein loading. **(F)** IL-6 induces phosphorylation of STAT3 in a dose dependent manner. HUH-7 cells (2×10^6^/ml) were treated with indicated concentrations of IL-6 (10 ng/ml) for 15 min and the whole-cell extracts were subjected to western blotting analysis. **(G)** Garcinol downregulates IL-6–induced phospho-STAT3 in HUH-7 cells. HUH-7 cells (2×10^6^/ml) were treated with 50 μM garcinol for the indicated durations and then stimulated with IL-6 (10 ng/ml) for 15 min and the whole-cell extracts were subjected to western blotting analysis. **(H)** Garcinol did not affect phospho-STAT3 (Ser^727^) levels in C3A cells. C3A cells (2×10^6^/ml) were treated with the indicated concentrations of garcinol for 6 h, and the whole cell extract was subjected to immune-blotting analysis using phospho-STAT3 (Ser^727^) antibody. The same blots were stripped and reprobed with STAT3 antibody to verify equal protein loading.

The polyisoprenylated benzophenone, garcinol is a potent natural compound, able to target multiple protein/enzymes thereby different pathways in the cellular system. These properties and the scaffold of garcinol have made garcinol an attractive molecule for anti-neoplastic therapeutics. We have investigated the effect of garcinol on constitutive and inducible STAT3 activation in HCC cells. We also have evaluated the effect of garcinol on various mediators of cellular proliferation, cell survival, and apoptosis. The structure of garcinol is shown in Figure 1A.

### Garcinol suppresses nuclear translocation of STAT3 and its phosphorylation

As nuclear translocation is central to the function of transcription factors [31], we intended to determine whether garcinol can modulate nuclear translocation of STAT3 and thereby affect its transcriptional role in cell. Interestingly, garcinol treatment inhibited the translocation of STAT3 to the nucleus in HCC cells as evident in our immunofluorescence assay (Figure 1B, Additional file 1: Figure S1, Additional file 2: Figure S2). Further, the ability of garcinol to modulate constitutive STAT3 activation in HCC cells was investigated. C3A cells were incubated with different concentrations of garcinol for 6 h, whole cell extracts were prepared and the phosphorylation of STAT3 was examined by Western blot analysis using antibodies which recognize STAT3 phosphorylation at tyrosine 705. Garcinol was found to inhibit the constitutive activation of STAT3 in C3A cells in a dose and time dependent manner as indicated by decreased phosporylated form of STAT3 in our immunoblotting experiment (Figure 1C and D). In dose dependent inhibition assay, maximum inhibition was observed at 50 μM concentration. The 50 μM garcinol could inhibit maximally the STAT3 phosphorylation at around 4–6 h of treatment. In JAK-STAT pathway, JAK2 is the kinase responsible for the phosphorylation of tyrosine 705 residue on STAT3. So, we wanted to verify the same under our experimental conditions with pharmacological modulation approach. To determine the effect of garcinol on JAK2 activation, C3A cells were treated for different time intervals with garcinol and phosphorylation of JAK2 was analyzed by Western blot. As shown in Figure 1E, JAK2 was constitutively active in C3A cells and pre-treatment with garcinol suppressed this phosphorylation in a time-dependent manner thereby suggesting that garcinol also targets the JAK2 kinase and inhibits the phosphorylation of STAT3.

Activation of STAT3 via phosphorylation has been demonstrated to be induced in response to various cytokines such as IL6 [32,33]. To investigate whether garcinol could modulate cytokine-induced STAT3 phosphorylation, HUH-7 cells (which have low levels of endogenous constitutively active STAT3) were activated with IL-6 and consequently effect of garcinol treatment was checked. Activation of STAT3 phosphorylation by IL-6 treatment was confirmed in a dose dependent assay (Figure 1F). Garcinol (50 μM) treatment for different time durations on IL-6 activated HUH-7 cells substantially suppressed STAT3 phosphorylation in a time-dependent manner (Figure 1G). Because STAT3 also undergoes phosphorylation at Ser 727, we next investigated whether garcinol has an effect on this phosphorylation. Interestingly, we found that garcinol had minimal effect on the STAT3 activation at Ser 727 residue, thereby suggesting that it specifically blocks tyrosine phosphorylation of STAT3 in C3A cells (Figure 1H). Collectively, these data indicate that garcinol suppresses STAT3 tyrosine phosporylation, thereby inhibiting its nuclear localization.

### Garcinol inhibits STAT3 dimerization via physical interaction as well as inhibition of acetylation

**Figure 2:**
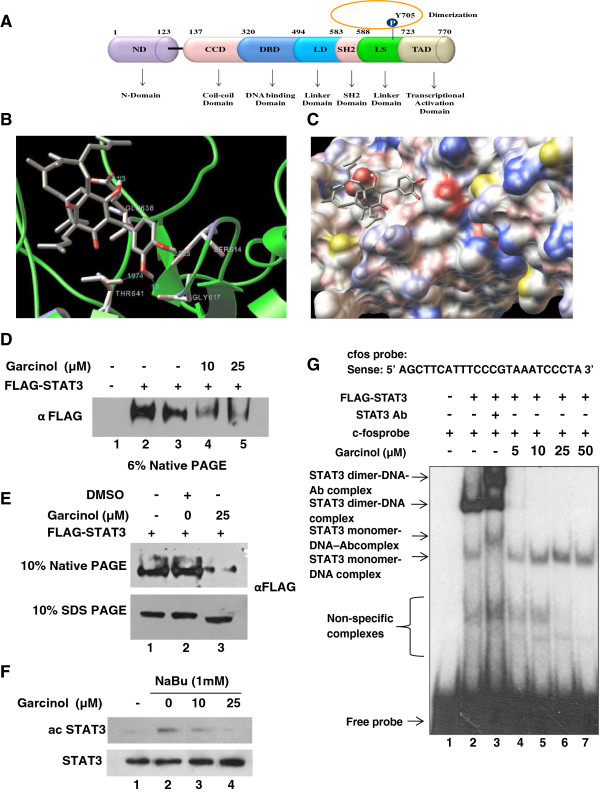
**Garcinol docks on to SH2 domain of STAT3 and inhibits STAT3 dimerization and acetylation, thereby preventing its nuclear localization and DNA binding. (A)** Diagrammatic representation of different domains of STAT3. **(B)** Predicted model of garcinol binding to the STAT3 SH2 domain as shown by computational docking. Protein structure information was obtained from Protein Data Bank entry 1BG1. Garcinol shows interaction with residues Ser614, Gly617, Glu638 and Thr641 of STAT3. **(C)** Surface view of docked garcinol on STAT3 protein surface. **(D)** Garcinol inhibits STAT3 dimerization *in vitro*. Native protein extracts prepared from 293 T cells transfected with FLAG-STAT3 were incubated with two different concentrations of garcinol for 15 min at 30°C and were loaded in 6% SDS free native PAGE. STAT3 dimers were visualized by immune-blotting with anti-FLAG antibody. **(E)** Garcinol inhibits STAT3 dimerization *in vivo*. 293 T cells transfected with FLAG-STAT3 were treated with DMSO or garcinol (25 μM) for 3 h and whole cell lysates were prepared. Twenty micrograms of protein per sample was loaded onto a 10% native PAGE gel, and electrophoresis was performed in the absence of SDS and subjected to immune-blotting analysis. **(F)** Garcinol inhibits acetylation of STAT3 in cells in a dose dependent manner. HepG2 cells were incubated with 1 mM sodium butyrate (NaBu) for 6 h followed by treatment with different doses of garcinol. Cells were lysed in Laemmli buffer and immunoblotting was performed using antibodies against acetylated STAT3 and total STAT3. **(G)** Garcinol mediated inhibition of STAT3 acetylation reduces DNA binding ability of STAT3. FLAG-STAT3 was immuno-affinity purified from HEK293T cells (after transient transfection of FLAG-STAT3 construct for 24 h) and was incubated with garcinol in a dose dependent manner and EMSA assay was carried out using a ^32^P-labelled oligo containing STAT3 binding site. For supershift validation, STAT3 antibody was added in lane 3.

STAT3 is a 770 amino acid long protein and the dimerization domain is positioned on its C-terminus (Figure 2A). STAT3 dimerization occurs through the interaction of the SH2 domain on one STAT3 molecule with a loop segment (from Ala702 to Phe716) on the other STAT3 monomer [34]. The phosphorylated Tyr705 on one STAT3 molecule is a critical residue for binding to a cavity on the SH2 domain of the other STAT3 protein [35]. To investigate whether garcinol can bind to the STAT3 SH2 domain, computational modeling was performed. The crystal structure of STAT3 at 2.25- Å resolution [34] was obtained from the Protein Data Bank (PDB ID code 1BG1) and used for computational docking analysis. The results of this modeling approach predicted multiple interactions of Garcinol with amino acids Ser 614, Gly 617, Glu 638 and Thr 641 on STAT3 (Figure 2B and C). The docking energy was - 4.45 kcal and inhibition constant was 543.21 μM. The intermolecular energy, internal energy and torsional energy was - 8.63 kcal, −3.21 kcal and 4.18 kcal respectively. The residues which were predicted to interact with garcinol fall in SH2 domain. SH2 domain being the interacting module for dimerization of STAT3, we hypothesized that garcinol might interfere with dimerization of the protein via direct binding. To experimentally validate the prediction, we transfected HEK293T cells with FLAG-STAT3 expression mammalian construct. After 24 h of transfection, cells were harvested and lysates were prepared in isotonic buffer. Two different concentrations of garcinol (10 and 25 μM) were incubated with the lysates (Figure 2D, lanes 4 and 5) and DMSO was added as the solvent control (Figure 2D, lane 3). We observed that garcinol could inhibit STAT3 dimerization in a dose dependent manner *in vitro* suggesting direct interaction of STAT3 and garcinol (Figure 2D, compare lane 3 versus lanes 4 and 5). To further validate the inhibition of STAT3 dimerization *in vivo*, we treated FLAG-STAT3 transfected HEK293T cells with garcinol (25 μM) and performed a native PAGE using the whole cell lysate and observed that garcinol could efficiently reduce the levels of dimer form of STAT3 in mammalian cells as well, which is consistent with the results, obtained from *in vitro* experiments (Figure 2E).

Apart from phosphorylation, acetylation of STAT3 also plays pivotal roles in its dimerization, DNA binding and promotion of tumorigenesis [13,14,36]. Overexpression of lysine acetyltransferases such as p300 has been documented in various cancers including HCC cells [37,38]. As garcinol is a well known lysine acetyltransferase inhibitor, we explored the possibility of inhibition of STAT3 acetylation by garcinol. We observed that garcinol could efficiently inhibit STAT3 acetylation in sodium butyrate pretreated HepG2 cells (Figure 2F). The dimer form of STAT3 is the most active form that has the ability to bind on the promoters of its target genes. The dimerization ability of STAT3 depends upon its phosphorylation and acetylation status [13]. Since garcinol inhibits both the acetylation and phosphorylation of STAT3 and furthermore presumably binds directly at the dimerization domain, garcinol treatment would inhibit the DNA binding of STAT3. We performed a DNA binding assay using oligonucleotides derived from *c-fos* promoter containing STAT3 binding site for this purpose. FLAG-STAT3 construct was transfected in HEK293T cells for 24 h and FLAG tagged STAT3 was pulled down using immunoaffinity chromatography and was used for EMSA. The DNA binding ability of STAT3 seemed to be abrogated because of inhibition of dimerization with incubation of garcinol *in vitro* which further led to concomitant increase in weak interaction of STAT3 monomer with the target DNA. This suggests the potential of garcinol to inhibit binding ability of STAT3 to its target promoters (Figure 2G).

### Garcinol suppresses STAT3 dependent transcription and downregulates the expression of cyclin D1, Bcl-2, Bcl-xL, Mcl-1, survivin, and VEGF thereby affecting cell cycle progression as well as cell viability

**Figure 3:**
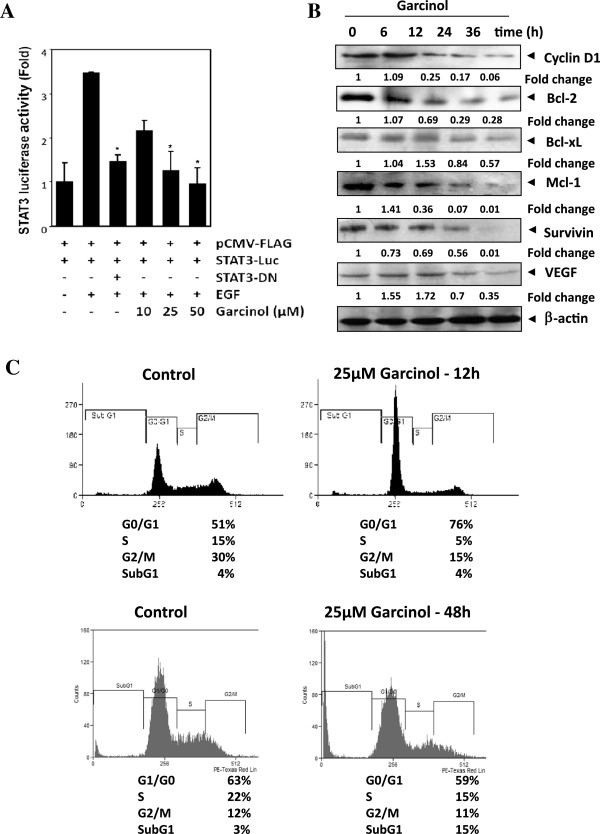
**Garcinol suppresses STAT3 regulated genes involved in proliferation, survival and angiogenesis*. *(A)** Garcinol inhibits EGF–induced STAT3-dependent reporter gene expression. PLC/PRF5 cells (5 × 10^5^/mL) were transfected with STAT3-luciferase (STAT3-Luc) plasmid, incubated for 24 h, and treated with indicated doses of garcinol for 4 h and then stimulated with EGF (100 ng/mL) for 2 h. Whole-cell extracts were then prepared and analyzed for luciferase activity. The results shown are representative of three independent experiments. * indicates p value <0.05. **(B)** Garcinol downregulates the expression of cyclin D1, Bcl-2, Bcl-xL, Mcl-1, survivin, and VEGF. C3A cells (2×10^6^/ml) were treated with 25 μM garcinol for indicated time intervals, after which whole-cell extracts were prepared and 30 μg portions of those extracts were subjected to western blotting, upon resolving on 10% SDS-PAGE. **(C)** Garcinol causes accumulation of cells in G0/G1 phase of cell cycle and enrichment in Sub G1 population. C3A cells (2×10^6^/mL) were treated with 25 μM garcinol for 12 h and 48 h, after which the cells were washed, fixed, stained with PI, and analyzed for DNA content by flow cytometry.

Our above results showed that garcinol perturbed phosphorylation and acetylation and also nuclear translocation of STAT3. As these are the key events dictating functional behavior of STAT3, we next determined whether garcinol affects STAT3-dependent gene transcription. When PLC/PRF5 cells, transiently transfected with the pSTAT3-Luc construct, were stimulated with EGF, STAT3-mediated luciferase gene expression was found to be substantially increased. Dominant-negative STAT3 significantly blocked this increase, indicating specificity. Remarkably, when the cells were pretreated with garcinol, EGF–induced transcriptional activity of STAT3 was inhibited in a dose-dependent manner (Figure 3A).

**Figure 4:**
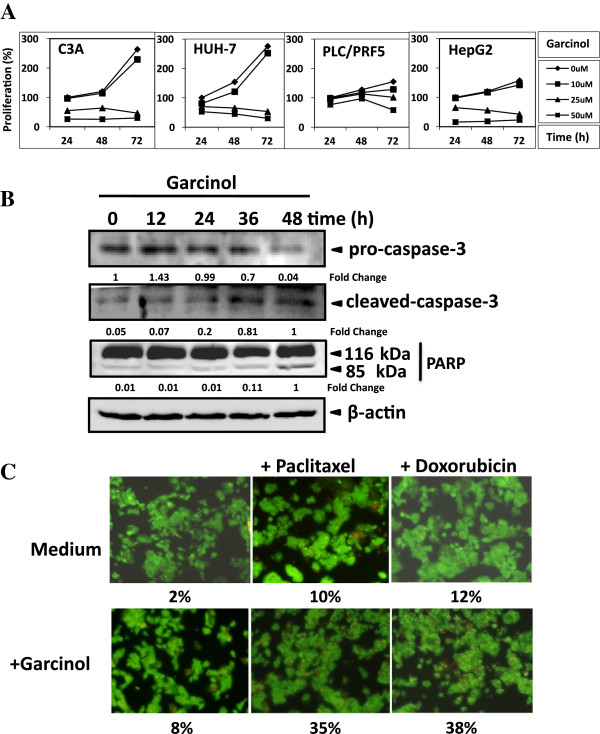
**Garcinol reduces cellular proliferation and induces apoptosis in HCC cells. (A)** Garcinol inhibits proliferation of HCC cells in a dose and time dependent manner. Different cell lines were plated in triplicate, treated with indicated concentrations of garcinol, and then subjected to MTT assay after 24, 48 and 72 h to analyze proliferation of cells. Data represents result of three independent experiments. **(B)** Garcinol promotes apoptosis: C3A cells were treated with 50 μM garcinol and the whole cell extracts were subjected to immune-blotting with antibodies against pro-caspase-3, cleaved caspase-3 and PARP taking β-actin as loading control. **(C)** Garcinol potentiates the apoptotic effect of doxorubicin and paclitaxel. C3A cells (1×10^6^/ml) were treated with 10 μM garcinol and 10 nM doxorubicin or 5 nM paclitaxel alone or in combination for 24 h at 37°C. Cells were stained with a Live/Dead assay reagent for 30 min and then analyzed under a fluorescence microscope as described in Materials and Methods.

STAT3 activation has been reported to regulate the expression of various gene products involved in cell survival, proliferation, angiogenesis and chemoresistance [8]. We found that expression of the cell cycle regulator *cyclin D1*, the antiapoptotic proteins *Bcl-2, Bcl-xL, survivin, Mcl-1* and the angiogenic gene product *VEGF* all of which have been reported to be regulated by STAT3 were modulated by garcinol treatment. Their expression decreased in a time-dependent manner, with maximum suppression observed at around 24–36 h (Figure 3B). Since Cyclin D1 is required for the progression of cells from the G1 phase of the cell cycle to S phase and rapid decline in levels of cyclin D1 was observed in garcinol treated cells; we wanted to determine the effect of garcinol on cell cycle phase distribution. We found that garcinol treatment caused increased accumulation of cell population in G0/G1 phase of the cell cycle (Figure 3C) when C3A cells were treated with 25 μM of garcinol for 12 h. Moreover, garcinol treatment for longer duration (48 h) led to enrichment of cells in Sub-G1 population, indicating incidence of apoptosis. Understandably, cell cycle arrest at G0/G1 phase as well as incidence of apoptosis would mean reduced cell proliferation which was reflected in our MTT assay. Garcinol inhibited the proliferation of C3A, HUH-7, PLC/PRF5, and HepG2 cells in a dose and time dependent manner (Figure 4A). To further validate and confirm that garcinol mediated inhibition of STAT3 activation is really inducing apoptosis mediated cell death, thereby contributing to reduced proliferation of HCC; C3A cells were treated with garcinol and checked for pro-caspase and cleaved-caspase levels in cells. There was a time-dependent activation of caspase-3 upon garcinol treatment as indicated from decrease in levels of pro-caspase 3 and increased levels of cleaved form of the protein as observed in our immunoblotting assay (Figure 4B). Activation of caspase-3 leads to the cleavage of 116 kDa PARP proteins into an 85 kDa fragment, which was confirmed in our immunoblotting experiment (Figure 4B). These results clearly suggest that garcinol induces caspase-3-dependent apoptosis in HCC cells.

There have been constant efforts to develop anti-cancer drugs to fight progression of different tumors in human. Among various chemotherapeutic agents, doxorubicin, an anthracycline antibiotic, and paclitaxel, a mitotic inhibitor, have been used for HCC treatment [39]. To look into druggable nature of garcinol we examined whether garcinol can potentiate the effect of these drugs in context of hepato-cellular carcinoma. C3A cells were treated with garcinol together with either doxorubicin or paclitaxel, and then apoptosis was measured by the live/dead assay. As shown in Figure 4C, garcinol significantly enhanced the apoptotic effects of paclitaxel from 10% to 35% and of doxorubicin from 12% to 38% respectively. Thus, our experimental results suggest that garcinol could act in combination with other anti-cancer agents to enhance apoptosis and retard cellular proliferation in HCC, therefore, making it amenable for drug development.

### Garcinol suppresses the growth of human HCC *in vivo* and STAT3 activation in tumor tissues

**Figure 5:**
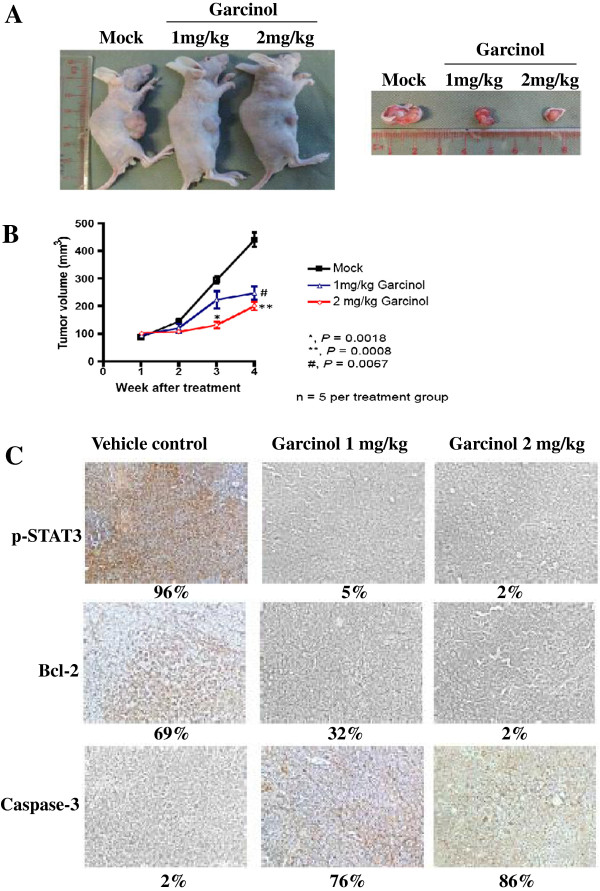
**Garcinol inhibits the growth of human HCC in vivo. (A)** Garcinol treatment reduced the size of tumor induced in nude mice. Athymic mice bearing subcutaneous PLC/PRF5 tumors were treated for 5 times a week for 4 consecutive weeks with 1 mg/kg and 2 mg/kg garcinol (n = 5) or corn oil alone (n = 4). Tumor size was measured and compared for different conditions. **(B)** Reduction in tumor volume upon garcinol treatment was statistically significant. * = P < 0.001 (One-way ANOVA). **(C)** Immunohistochemical analysis showed the inhibition in expression of phospho-STAT3, and Bcl-2 and increased levels of caspase-3 expression in garcinol treated mice tissues as compared with control group. Percentage indicates positive staining for the given biomarker. The photographs were taken at the magnification of × 20.

To investigate the anti-tumor potential of garcinol *in vivo,* a subcutaneous model of human HCC was generated in athymic mice via intra-peritoneal injection of PLC/PRF5 cells. Garcinol at doses of 1 mg/kg and 2 mg/kg induced significant inhibition of tumor growth compared with the DMSO-treated controls (Figure 5A). Two-way repeated measures ANOVA showed a statistically significant difference in tumor growth between the garcinol-treated and control groups (Figure 5B). We further evaluated the effect of garcinol on constitutive p-STAT3 levels in HCC tumor tissues by immunohistochemical analysis and found that garcinol substantially inhibited the constitutive STAT3 activation in treated group as compared with control group (Figure 5C). The effect of garcinol was also analyzed on the expression of Bcl-2 (marker of survival) and caspase-3 (marker of apoptosis). As shown in Figure 5C, expression of Bcl-2 was downregulated and that of caspase-3 was substantially increased in garcinol treated group as compared with control group, indicating incidence of cell death via apoptosis.

Taken together, these data suggest that garcinol, being able to inhibit phosphorylation and acetylation of STAT3, can impair its nuclear localization and DNA binding ability, thereby negatively regulate transcriptional activation ability of STAT3 and ultimately lead to reduced proliferation as well as induction of apoptosis in HCC; therefore, could be exploited as a potential antineoplastic therapeutic in HCC.

## Discussion

The aim of this study was to determine whether garcinol exerts its anti-cancer effects in HCC cells through the abrogation of the STAT3 signaling pathway, targeting the post-translational modification of STAT3 and dimerization of the transcription factor. We found that this benzophenone inhibited both constitutive and inducible STAT3 activation in human HCC cells concomitant with the inhibition of phosphorylation and acetylation of STAT3 and thereby the dimerization and its DNA binding ability. As a consequence garcinol further downregulated the expression of various STAT3-regulated genes including, *cyclin D1, Bcl-2, Bcl-xL, survivin, Mcl-1,* and *VEGF*, caused the inhibition of proliferation, and induced substantial apoptosis in HCC cells. We subsequently investigated the therapeutic potential of garcinol in HCC xenograft grown in mouse model. Intra-peritoneal injection of garcinol into nude mice bearing subcutaneous PLC/PRF5 xenografts resulted in significant suppression of tumor progression and suppression of expression of p-STAT3 in garcinol treated tumor tissues.

Using computational modeling, we found that garcinol may directly bind to the SH2 domain of STAT3. Computational modeling also showed the mode of interaction through which garcinol interacts with amino acids Ser 614, Gly 617, Glu 638 and Thr 641 residues of STAT3. These observations suggested that garcinol may inhibit the dimerization of STAT3 through its interaction with the SH2 pocket of STAT3. By employing high-throughput screening, recently, another natural compound- cryptotanshinone, a benzofuran, has been identified, which also presumably binds to the SH2 domain of STAT3 and inhibit its dimerization [40]. Our finding does strengthen this hypothesis further that the systemic blocking of STAT3 dimerization through the small molecule mediated intervention could be a useful tool to suppress the constitutive activation of STAT3. However, unlike garcinol, the cryptotanshinone inhibit the STAT3 phosphorylation in a JAK2 independent manner [14]. We observed that garcinol could also suppress IL-6 -induced STAT3 activation in HCC cells and these effects of garcinol correlated with the suppression of upstream protein tyrosine kinase JAK2 autophosphorylation. Previous studies have indicated that the IL-6/ gp130/JAK signaling pathway can phosphorylate STAT3 Tyr705 in various tumor cells, including HCC [41,42]. We also observed that garcinol suppressed nuclear translocation of STAT3 and induction of EGF-induced reporter activity by STAT3.

Apart from the phosphorylation, STAT3 is also subjected to acetylation which is pivotal for its stable dimer formation and sequence specific DNA binding [13,36]. Furthermore, HCC is also associated with hyperacetylation of histones and non-histone proteins, which contributes to the cancer progression. In consistent with the previous reports about garcinol and its acetyltransferase inhibitory activity [43,44]; we observed that garcinol could effectively inhibit STAT3 acetylation in a dose dependent manner. Our studies indicated that inhibition of STAT3 acetylation impaired its nuclear localization and DNA binding. This suggests that garcinol could manifest its effect on STAT3 activation through multiple mechanism(s) especially by inhibiting phosphorylation and acetylation of STAT3 and directly binding to its SH2 domain.

STAT3 phosphorylation plays a crucial role in proliferation and survival of tumor cells. Various types of cancer, including head and neck cancers [45] multiple myeloma [46], lymphomas, and leukemia [47], also have constitutively active STAT3. The suppression of constitutively active STAT3 in HCC cells raises the possibility that garcinol might also suppress constitutively activated STAT3 in other types of cancer cells. Previously, it has been reported that garcinol can suppress NF-κB activation in the breast and pancreatic cancer cells [28,48]. Interestingly, a recent report indicated that STAT3 can prolong NF-κB nuclear retention through acetyltransferase p300-mediated RelA acetylation, thereby interfering with NF-κB nuclear export [49]. Thus, garcinol mediated inhibition of acetyltransferase activity, as well as STAT3 phosphorylation by JAK2 may synergistically affect transcriptional activity of NF-κB and thereby act as an anti-neoplastic compound.

We also observed that garcinol can suppress the expression of several STAT3-regulated genes; including proliferative (*cyclin D1*), antiapoptotic genes (*Bcl-2, Bcl-xL, survivin, and Mcl-1*) and angiogenic gene (*VEGF*). The downregulation of the expression of *Bcl-2, Bcl-xL, survivin* and *Mcl-1* is likely linked with the garcinol’s ability to induce apoptosis in HCC cells as clearly evident by activation of pro-caspase-3 and cleavage of PARP. The down modulation of *VEGF* expression as reported here may also explain the anti-angiogenic potential of this benzophenone but needs further investigation.

Whether these *in vitro* observations with garcinol have any relevance to the *in vivo* scenario was also investigated. Our results clearly showed that garcinol significantly suppressed HCC growth in nude mouse model, downregulated the expression of phospho-STAT3 and Bcl-2, and increased the levels of caspase-3 in treated group as compared to control group. Additionally, we did not notice any observable adverse effects in animals following treatment with garcinol although we did not perform a detailed preclinical toxicological analysis in this study. Moreover, to the best of our knowledge, no prior studies with garcinol in xenograft HCC models have been reported so far, and our overall findings suggest that garcinol has a tremendous potential to serve as a lead molecule for the treatment of HCC through the modulation of STAT3 activation pathway. Two recent reports on garcinol mediated inhibition of growth of xenografted tumor developed from different cancer sources in the context of STAT3 function further underscore the therapeutic implications of garcinol [45,50].

Garcinol has been shown to be well tolerated in pre-clinical studies using various cancer models, with no reported toxicity so far [51-53]. Garcinol as such has never been tested in humans before and hence its clinically relevant doses are not known as yet. Thus, overall, our *in vitro* and *in vivo* findings clearly demonstrate that the anti-proliferative and apoptotic effects of garcinol in HCC are mediated through suppression of STAT3 activation and provide a sound basis for pursuing the use of garcinol to overcome toxicity and enhance treatment efficacy for HCC patients.

## Conclusion

Our results show that garcinol could suppress constitutive and inducible activation of STAT3 in HCC. Garcinol could inhibit STAT3 phosphorylation and acetylation, thereby prevent its dimerization as well as nuclear localization. As a consequence garcinol further led to downregulation of expression of various STAT3-regulated genes including, *cyclin D1, Bcl-2, Bcl-xL, survivin, Mcl-1,* and *VEGF* and caused the inhibition of proliferation and induced substantial apoptosis in HCC cells. Finally, garcinol suppressed growth of HCC in xenograft mice model, underscoring its importance as an anti-cancer therapeutic agent.

## Materials and methods

### Reagents

Garcinol with purity greater than 98% was prepared from *Garcinia indica* fruit rind as described previously [15]. A 50 mM stock solution of garcinol was prepared in dimethyl sulfoxide and stored at −20°C to be used within 3 months after preparation. The stored solution was diluted with RPMI 1640 medium and further diluted in cell culture medium to make working concentrations. Hoechst 33342, MTT, Tris, glycine, NaCl, SDS, BSA, and EGF were purchased from Sigma-Aldrich (St. Louis, MO). RPMI 1640, fetal bovine serum (FBS), 0.4% trypan blue vital stain, and antibiotic-antimycotic mixture were obtained from Invitrogen (Carlsbad, CA, USA). Rabbit polyclonal antibodies against STAT3 and mouse monoclonal antibodies against phospho- STAT3 (Tyr 705), STAT3 (Ser 727), Bcl-2, Bcl-xL, cyclin D1, survivin, Mcl-1, VEGF, caspase-3, cleaved caspase-3, and PARP were obtained from Santa Cruz Biotechnology (Santa Cruz, CA). Antibodies for phospho-specific JAK2 (Tyr 1007/1008) and JAK2 were purchased from Cell Signaling Technology (Beverly, MA). FLAG antibody was purchased from Sigma-Aldrich (St. Louis, MO). Acetylated STAT3 (Lys-685) antibody was obtained from Thermo Scientific Pierce (Thermo Scientific Pierce, USA). Goat anti-rabbit-horse radish peroxidase (HRP) conjugate and goat anti-mouse HRP were purchased from Sigma-Aldrich (St. Louis, MO). Bacteria-derived recombinant human IL-6 was purchased from ProSpec-Tany TechnoGene Ltd. (Rehovot, Israel).

### Cell lines

Human hepatocellular carcinoma cell lines (C3A, HepG2, PLC/PRF5, and HUH-7) were obtained from American Type Culture Collection (Manassass, VA). C3A, HepG2, and HUH-7 cells were cultured in Dulbecco’s Modified Eagle Medium (DMEM) containing 1 X antibiotic-antimycotic solution with 10% FBS. PLC/PRF5 cells were cultured in DMEM containing 1x penicillin-streptomycin solution, non-essential amino acids, sodium pyruvate, and L-glutamine with 10% FBS. HEK293T also obtained from ATCC cells were cultured in Dulbecco’s Modified Eagle Medium (DMEM) containing 1 X antibiotic streptomycin solution, sodium pyruvate with 10% FBS.

### Western blotting

For detection of phopho-proteins, garcinol-treated whole-cell extracts were lysed in lysis buffer (20 mM Tris (pH 7.4), 250 mM NaCl, 2 mM EDTA (pH 8.0), 0.1% Triton X-100, 0.01 mg/ml aprotinin, 0.005 mg/ml leupeptin, 0.4 mM PMSF, and 4 mM NaVO_4_). Lysates were then spun at 14,000 rpm for 10 min to remove insoluble material and resolved on a 7.5% SDS-PAGE gel. After electrophoresis, the proteins were electrotransferred to a nitrocellulose membrane, blocked with Blocking One (Nacalai Tesque, Inc.), and probed with various phospho-antibodies (1:1000) overnight at 4°C. The blot was washed, exposed to HRP-conjugated secondary antibodies for 1 h, and finally examined by chemiluminescence (ECL; GE Healthcare, Little Chalfont, Buckinghamshire, UK).

To detect STAT3-regulated proteins, caspase-3 and PARP, C3A cells (2×10^6^/ml) were treated with garcinol for the indicated time. The cells were then washed and the protein was extracted by incubation for 30 min on ice in 0.05 ml buffer containing 20 mM HEPES, pH 7.4, 2 mM EDTA, 250 mM NaCl, 0.1% NP-40, 2 μg/ml leupeptin, 2 μg/ml aprotinin, 1 mM PMSF, 0.5 μg/ml benzamidine, 1 mM DTT, and 1 mM sodium vanadate. The lysate was centrifuged and the supernatant was collected. Whole-cell extract protein (30 μg) was resolved on 12% SDS-PAGE, electrotransferred onto a nitrocellulose membrane, blotted with various antibodies and then detected by chemiluminescence (ECL; GE Healthcare, Little Chalfont, Buckinghamshire, UK). The densitometric analysis of scanned blots was done using Image J software and the results are expressed as fold change relative to the control.

### Immunocytochemistry for STAT3 localization

C3A cells were plated in chamber slides in DMEM containing 10% FBS and allowed to adhere for 24 h. On next day, following treatment with garcinol for 4 h, the cells were fixed with cold acetone for 10 min, washed with PBS and blocked with 5% normal goat serum for 1 h. The cells were then incubated with rabbit polyclonal anti-human STAT3 antibody (dilution, 1/100). After overnight incubation, the cells were washed and then incubated with goat anti-rabbit IgG-Alexa 594 (1/100) for 1 h and counterstained for nuclei with Hoechst (50 ng/ml) for 5 min. Stained cells were mounted with mounting medium (Sigma-Aldrich) and analyzed under an fluorescence microscope (Olympus DP 70, Japan).

### Docking analysis

The Mus musculus STAT3 Protein structure (UniProtKB/Swiss-Prot id P42227) was downloaded from PDB, (PDB id 1bg1) having resolution 2.25 Å. It is a phosphorylated monomer structure with chain A (consisting of 558 amino acids: 136–716), which is complexed with a DNA chain (designated as chain B). The DNA chain was removed from the complex while preparing this molecule for docking.

Protein was prepared in Autodock 1.5.4. The water molecules and DNA binding structure were removed and then polar hydrogen and Gasteiger charges were added to it. Grid box was prepared at its SH2 domain having dimensions - X:116, Y: 82 and Z: 122., keeping the grid spacing at 0.375. Coordinate file of ligand garcinol was created using Gaussian 03 program. The ligand was then prepared using Autodock 1.5.4 tool, where Gasteiger charges and polar hydrogen were added and number of torsions were set for each. Docking was carried out using Lamarckism genetic algorithm method.

### STAT3 luciferase reporter assay

PLC/PRF5 cells were plated in ninety six-well plates with 1 x 10^4^ per well in DMEM containing 10% FBS. The STAT3-responsive elements linked to a luciferase reporter gene were transfected with wild-type STAT3 or dominant-negative STAT3-Y705F (STAT3F). These plasmids were a kind gift from Dr. Bharat B. Aggarwal at M D Anderson Cancer Center, Houston, TX. Transfections were done according to the manufacturer's protocols using Fugene-6 obtained from Roche (Indianapolis, IN). At 24 h post-transfection, cells were pretreated with garcinol for 4 h and then induced by EGF for additional 2 h before being washed and lysed in luciferase lysis buffer from Promega (Madison, WI, USA). Luciferase activity was measured with a luminometer by using a luciferase assay kit (Promega) and was normalized to β-galactosidase activity. All luciferase experiments were done in triplicate and repeated three or more times.

### MTT assay

The anti-proliferative effect of garcinol against HCC cells was determined by the MTT dye uptake method as described previously [11]. Briefly, the cells (5x10^3^/ml) were incubated in triplicate in a 96-well plate in the presence or absence of indicated concentration of garcinol in a final volume of 0.2 ml for different time intervals at 37°C. Thereafter, 20 μl of MTT solution (5 mg/ml in PBS) was added to each well. After a 2-h incubation at 37°C, 0.1 ml of lysis buffer (20% SDS, 50% dimethylformamide) was added; incubation was continued overnight at 37°C; and then the optical density (OD) at 570 nm was measured by Tecan plate reader.

### Live/Dead assay

Apoptosis of cells was determined by Live/Dead assay kit (Life technologies, Carlsbad, USA) that measures intracellular esterase activity and plasma membrane integrity as described previously [11]. Briefly, 1 X10^6^ cells were incubated with garcinol/doxorubicin/ paclitaxel alone or in combination for 24 h at 37°C. Cells were stained with the Live/Dead reagent (5 μM ethidium homodimer, 5 μM calcein-AM) and then incubated at 37°C for 30 min. Cells were analyzed under a fluorescence microscope (Olympus DP 70, Japan).

### Cell cycle analysis

To determine the effect on the cell cycle, C3A cells were treated with garcinol (25 μM) for 12 h and 48 h. Thereafter cells were washed, fixed with 70% ethanol. Cells were then washed, resuspended, and stained in PBS containing 10 μg/ml propidium iodide (PI) and 1 μg/ml RNase A in PBS for 30 min at room temperature. Cell distribution across the cell cycle was analyzed with a CyAn ADP flow cytometer (Dako Cytomation).

### In vitro dimerization assay

Whole cell extracts of HEK293T cells containing FLAG-STAT3 were prepared using ice-cold isotonic buffer [20 mmol/L Tris (pH 7.0), 150 mmol/L NaCl, 6 mmol/L MgCl2, 0.8 mmol/L PMSF, and 20% glycerol]. Homogenization of lysates was performed using a 30-gauge syringe and then cleared by centrifugation at 13,000 rpm for 30 min at 4°C. Equal amounts of lysates were incubated with increasing amounts of garcinol with DMSO as solvent control at 30°C for 15 min. STAT3 dimers were identified upon loading the treated lysates in a 10% SDS free PAGE gel and immunblotted using FLAG antibody.

### Electrophoretic mobility shift assay

Oligonucleotide derived from c-fos promoter containing STAT3 binding sites was end labeled with [γ-^32^P] ATP by T4 polynucleotide kinase (Merck Biosciences). Labeled strand was then annealed with its complementary unlabelled strand for the EMSA studies. FLAG-STAT3 was affinity purified from HEK293T cells after transient transfection of FLAG-STAT3 construct for 24 h and was incubated with labeled oligo along with appropriate concentration of garcinol for 20 min at 30°C. For supershift validation, STAT3 antibody was added in the appropriate sample and was incubated for another 20 min at 30°C. The complexes were then run in a 5% native PAGE using 0.5X Tris-borate-EDTA buffer. Gels were then dried and exposed for autoradiography in a phosphoimager cassette.

### Tumor model

All procedures involving animals were reviewed and approved by NUS Institutional Animal Care and Use Committee. Six week-old athymic nu/nu female mice (Animal Resource Centre, Australia) were implanted subcutaneously in the right flank with (3 × 10^6^ PLC/PRF5 cells/100 μl saline). When tumors have reached 0.5 cm in diameter, mice received intra-peritoneal injection of 1 mg/kg and 2 mg/kg garcinol in 200 μl corn oil (n = 5) or corn oil alone (n = 5), 5 doses per week for 3 consecutive weeks. Animals were euthanized at day 22 after first therapeutic dose injection. Tumor dimensions were measured using a digital caliper, and the tumor volume (V) calculated using the formula: V=π/6 X length X (width) 2. Growth curves were plotted using average relative tumor volume within each experimental group at the set time points.

### Immunohistochemical (IHC) analysis of tumor samples

Solid tumors from control and garcinol treated mice were fixed with 10% phosphate buffered formalin, processed and embedded in paraffin. Sections were cut and deparafinized in xylene, and dehydrated in graded alcohol and finally hydrated in water. Antigen retrieval was performed by boiling the slide in 10 mM sodium citrate (pH 6.0) for 30 min. Immunohistochemistry was performed following manufacturer instructions (DAKO LSAB kit).

### Confocal Laser Microscopy

HepG2 cells were grown on polylysine-coated coverslips for 24 h in the presence of Modified Eagle medium supplemented with 10% fetal bovine serum. Appropriate treatments were given to cells. Then cells were washed with 1XPBS, fixed with 4% paraformaldehyde and permeabilized with 0.5% Triton X-100. Anti-STAT3 (Abcam) antibody was used as primary antibody and fluorescently labeled anti-Rabbit Alexa-488 conjugated (Invitrogen) was used as secondary antibody. Hoechst and Alexa fluorescence were visualized using a Carl Zeiss confocal laser scanning microscope (Axioskop 2 Plus).

### Statistical analysis

Statistical analysis was performed by Student’s t-test and one way analysis of variance, (ANOVA). A p value of less than 0.05 was considered statistically significant.

## Abbreviations

STAT3: Signal transducer and activator of transcription 3; HCC: Hepatocellular carcinoma; FBS: Fetal bovine serum; MTT: 3-(4, 5-dimethylthiazol-2-yl)-2, 5-diphenyl-2H-tetrazolium bromide; JAK: Janus-like kinase.

## Competing interests

The authors declare that they have no competing interests.

## Authors’ contributions

JB has done isolation and characterization of garcinol. NN has done collection and authentication of natural material. GS was involved in designing and execution as well as supervision of work. SC, PS, AKB, GS were involved in cell line based experiments, data analysis, also in the preparation of manuscript. PR, FL, MKS, KFW, APK, and KMH were involved in different cell and animal based experiments. JML and TKK have done overall supervision of work. All authors read and approved the final version of manuscript.

## Authors’ information

GS and SC are joint first authors.

## Supplementary Material

Additional file 1: Figure S1 Garcinol treatment suppressed nuclear translocation of STAT3 in hyperacetylation background. HepG2 cells grown on poly-lysine coated coverslips were incubated with 1mM NaBu for 6hrs to induce the internal acetylation. Cells were then treated with two different concentrations of garcinol (10μM and 25 μM) for 4hrs and processed for confocal imaging using antibody against STAT3. Click here for file

Additional file 2: Figure S2 Garcinol treatment suppressed nuclear translocation of STAT3 in HepG2 cells: HepG2 cells were grown on poly-lysine coated coverslips. Cells were then treated with either DMSO or 10μM of garcinol for 3h and processed for confocal imaging after immune-staining with antibody against STAT3. Click here for file
