# Supplementary material for: Inhibition of STAT3 dimerization and acetylation by garcinol suppresses the growth of human hepatocellular carcinoma in vitro and in vivo
Source: Mol Cancer. 2014 Mar 21;13:66. doi: 10.1186/1476-4598-13-66 (PMC3998115; doi:10.1186/1476-4598-13-66)
Supplement: Additional file 1: Figure S1 — Garcinol treatment suppressed nuclear translocation of STAT3 in hyperacetylation background. HepG2 cells grown on poly-lysine coated coverslips were incubated with 1mM NaBu for 6hrs to induce the internal acetylation. Cells were then treated with two different concentrations of garcinol (10μM and 25 μM) for 4hrs and processed for confocal imaging using antibody against STAT3. [file 1476-4598-13-66-S1.docx]

**Results:**

**Additional file 1: Figure S1:**

**Figure S1: Garcinol treatment suppressed nuclear translocation of STAT3 in hyperacetylation background.** HepG2 cells grown on poly-lysine coated coverslips were incubated with 1mM NaBu for 6hrs to induce the internal acetylation. Cells were then treated with two different concentrations of garcinol (10μM and 25 μM) for 4hrs and processed for confocal imaging using antibody against STAT3.
