# Supplementary material for: Inhibition of STAT3 dimerization and acetylation by garcinol suppresses the growth of human hepatocellular carcinoma in vitro and in vivo
Source: Mol Cancer. 2014 Mar 21;13:66. doi: 10.1186/1476-4598-13-66 (PMC3998115; doi:10.1186/1476-4598-13-66)
Supplement: Additional file 2: Figure S2 — Garcinol treatment suppressed nuclear translocation of STAT3 in HepG2 cells: HepG2 cells were grown on poly-lysine coated coverslips. Cells were then treated with either DMSO or 10μM of garcinol for 3h and processed for confocal imaging after immune-staining with antibody against STAT3. [file 1476-4598-13-66-S2.docx]

**Results:**

**Additional file 2: Figure S2:**


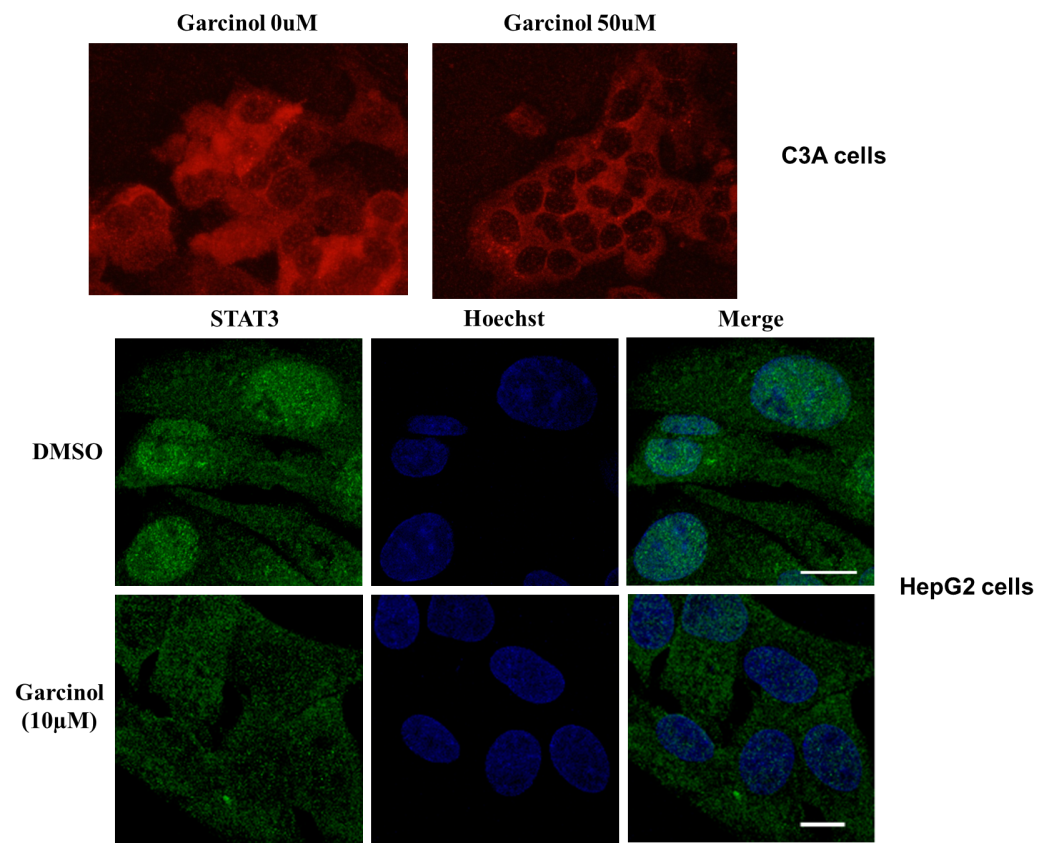


**Figure S2: Garcinol treatment suppressed nuclear translocation of STAT3 in HepG2 cells:** HepG2 cells were grown on poly-lysine coated coverslips. Cells were then treated with either DMSO or 10μM of garcinol for 3h and processed for confocal imaging after immune-staining with antibody against STAT3.
